# Regulation of Tissue Fibrosis by the Biomechanical Environment

**DOI:** 10.1155/2013/101979

**Published:** 2013-05-28

**Authors:** Wayne Carver, Edie C. Goldsmith

**Affiliations:** Department of Cell Biology and Anatomy, University of South Carolina, School of Medicine, Columbia, SC 29209, USA

## Abstract

The biomechanical environment plays a fundamental role in embryonic development, tissue maintenance, and pathogenesis. Mechanical forces play particularly important roles in the regulation of connective tissues including not only bone and cartilage but also the interstitial tissues of most organs. *In vivo* studies have correlated changes in mechanical load to modulation of the extracellular matrix and have indicated that increased mechanical force contributes to the enhanced expression and deposition of extracellular matrix components or fibrosis. Pathological fibrosis contributes to dysfunction of many organ systems. A variety of *in vitro* models have been utilized to evaluate the effects of mechanical force on extracellular matrix-producing cells. In general, application of mechanical stretch, fluid flow, and compression results in increased expression of extracellular matrix components. More recent studies have indicated that tissue rigidity also provides profibrotic signals to cells. The mechanisms whereby cells detect mechanical signals and transduce them into biochemical responses have received considerable attention. Cell surface receptors for extracellular matrix components and intracellular signaling pathways are instrumental in the mechanotransduction process. Understanding how mechanical signals are transmitted from the microenvironment will identify novel therapeutic targets for fibrosis and other pathological conditions.

## 1. Introduction

Mechanical forces play integral roles in embryonic development, homeostasis, and pathogenesis. All cells in multicellular organisms are exposed to mechanical forces of varying degrees. Endothelial cells, for instance, are exposed to shear stress due to the passage of fluid through the cardiovascular system. Chondrocytes and other cells in joints are exposed to repetitive compressive forces. The effects of mechanical forces on cells and tissues have received greater attention as models have been developed to systematically analyze these effects. Many of the early studies in this regard were focused on cells and tissues that are influenced by obvious mechanical force including the cardiovascular and musculoskeletal systems. Early investigations in the mechanobiology field relied on relatively simple and imprecise systems. For instance, studies have utilized a hanging-drop culture system to examine the effects of tensile forces on connective tissue cells [1]. As interest grew in the mechanobiology field, innovative systems were developed to apply tensile strain to rat calvarial cells cultured on ribbons of collagen [2] and compressive forces to chick long bones [3].

The mechanobiology field began to move forward rapidly as *in vitro* model systems were developed to more precisely isolate the effects of mechanical forces on cellular processes. Various systems were engineered to apply uniaxial or multiaxial distension or stretch to cells grown on deformable substrata. These systems date back several decades to studies conducted on smooth muscle cells that were cultured on deformable elastin matrices [4, 5]. Among other responses, these studies illustrated a role for mechanical force in the growth and maintenance of skeletal and cardiovascular cells [6–9]. It has become increasingly clear that many aspects of cell behavior can be modulated by mechanical force including cell proliferation, differentiation, migration, and gene expression. The realization that most cells respond to mechanical stimuli has resulted in enhanced interest in the contribution of these forces to pathogenesis including tissue fibrosis and in the mechanisms whereby cells detect and respond to these forces.

Studies by Leung et al. [5] were among the first to illustrate that cyclic mechanical loading promotes the production of extracellular matrix (ECM) components by vascular smooth muscle cells. The ECM is a dynamic network composed primarily of collagens, noncollagenous glycoproteins, and proteoglycans. The ECM was historically appreciated for its function as a three-dimensional scaffold that played an essential role in tissue development and function. Alterations in ECM composition, organization, and accumulation can deleteriously impact embryonic development and organ homeostasis in adults. For instance, deficits in collagen production result in vascular weakness and aneurysms [10]. On the other extreme, increased accumulation of ECM components or fibrosis results in dysfunction of many organs.

The expression of ECM components is regulated by diverse biochemical factors including growth factors, cytokines, and hormones (see [11, 12] for recent reviews). In addition, ECM production can be modulated by electrical and mechanical stimuli. Until relatively recently, the role of mechanical forces in regulating gene expression and cell behavior has received little attention. This has changed as it has been realized that all cells are exposed to mechanical forces, and with the advent of *in vitro* testing systems the effects of these forces and the mechanisms of their actions have been and continue to be investigated.

## 2. Mechanical Stretch and Promotion of Tissue Fibrosis

Cells can be exposed to diverse types of extrinsic mechanical forces including mechanical stretch (tension), compression, and shear stress. A number of early studies utilized cells cultured on deformable membranes to examine the cellular effects of mechanical stretch. These studies illustrated that mechanical stretch of isolated cells mimicked many of the responses that had been characterized to increased load *in vivo*. For instance, mechanical stretch of skeletal myotubes elicited a hypertrophic response that included increased general protein synthesis and enhanced accumulation of contractile proteins [13]. 

Alterations in mechanical load *in vivo* had been known for some time to impact synthesis and deposition of the ECM. For instance, increased cardiovascular load has for some time been correlated to increased deposition of ECM components. The period immediately after birth is associated with increased cardiovascular load and rapid growth of the heart [14]. This period of “physiological hypertrophy” is also associated with rapid deposition and organization of ECM components, particularly interstitial collagens [15–18]. Increased mechanical load as seen during aortic constriction or stenosis also promotes myocardial hypertrophy and fibrosis in the adult heart [19, 20]. While a number of mechanical stretch devices have been utilized to mimic changes in mechanical forces seen *in vivo*, all have generally illustrated that mechanical stretch of matrix-producing cells (largely fibroblasts and smooth muscle cells) results in increased production of ECM components or a profibrotic response [21–24]. 

To more accurately mimic the *in vivo* environment, apparatuses are being developed to investigate mechanical forces in three-dimensional *in vitro* systems. Several recent studies have applied mechanical loads to cells cultured in three-dimensional scaffolds [25, 26]. The use of three-dimensional constructs provides important insight into the effects of complex mechanical forces on tissue properties, and the development of systems to apply and analyze mechanical load to these constructs will also be advantageous to efforts to engineer functional tissue constructs.

## 3. Effects of Tissue Stiffness on Fibrosis

While two-dimensional *in vitro* systems have been invaluable in elucidating the effects of mechanical forces on cells and the mechanisms of mechanotransduction, cells function within a three-dimensional environment whose mechanical properties can change during development [27] or various pathological conditions including fibrosis [28, 29], cancer [30–34], and atherosclerosis [35]. Due to accumulation of ECM components and cross-linking of these components, alterations in tissue stiffness are a common feature of fibrosis. For instance, pathological scars are stiffer relative to unwounded normal skin and typically consist of thicker collagen bundles [36]. Accumulation of ECM components alters the tissue mechanical properties, which in turn can deleteriously impact organ function [37]. Component cells sense and respond to ECM rigidity, which can regulate cell growth [38], shape [39], migration [40, 41], and differentiation [42, 43].

Seminal studies by Mauch et al. [44] were among the first to evaluate the effects of the biomechanical microenvironment on the expression of ECM components. The expression of ECM components and ECM-modifying enzymes was compared between cells cultured on tissue culture plastic, a rigid substratum, and three-dimensional collagen gels, a more flexible substratum. These studies illustrated that collagen expression is markedly decreased in fibroblasts cultured in three-dimensional collagen scaffolds compared to cells grown on tissue culture plastic. This effect was, at least in part, regulated at the mRNA level as *α*1(I), *α*2(I), and *α*1(III) collagen mRNAs were diminished in cells cultured in the three-dimensional scaffolds. Further studies by this group of investigators illustrated that collagenase activity is enhanced by culture in three-dimensional scaffolds promoting a collagenolytic phenotype in the less rigid environment of the collagen gels [45]. A number of studies have subsequently supported the concept that matrix rigidity propagates the profibrotic response. Culture of human colon fibroblasts on matrices that mimic the mechanical properties of the normal colon or the pathologically stiff colon of Crohn's disease patients demonstrated enhanced expression of ECM components and increased proliferation of fibroblasts on the stiffer matrix [46]. Similarly, culture of human dermal fibroblasts in collagen gels that were made stiffer by prestraining resulted in enhanced expression of collagen by dermal fibroblasts relative to that in unstrained scaffolds [47]. Liu et al. [48] have utilized a novel photopolymerization approach to generate polyacrylamide scaffolds with stiffness gradients that span the range of normal and fibrotic lung tissue (0.1 to 50 kPa). In this system, proliferation of lung fibroblasts was induced by increased scaffold stiffness. In contrast, matrix stiffness protected cells from apoptosis in response to serum starvation. The patterns of collagen *α*1(I) and *α*1(III) mRNA expression paralleled proliferation with increasing expression in stiffer regions of the scaffold. The expression of prostaglandin, which is an endogenous antifibrotic factor, was opposite to that of the collagens with increased levels in the less rigid portions of the construct. These studies and others indicate that the biomechanical properties of the microenvironment can direct the expression of ECM components and ECM-modifying enzymes with stiffer tissue properties contributing to enhanced ECM production. Less rigid matrices appear to promote an anti-fibrotic environment that includes increased production of matrix-degrading proteases and anti-fibrotic agents like prostaglandin.

Matrix rigidity impacts not only the expression of ECM components but also other parameters associated with fibrosis including the deposition and organization of these components. Studies by Halliday and Tomasek [49] illustrated that fibroblasts cultured in stabilized three-dimensional collagen gels generate stress that is transmitted throughout the collagen scaffold. These cells develop large actin microfilament bundles and organize fibronectin into extracellular fibrils. Fibroblasts cultured in free-floating collagen gels generate less stress and lack fibronectin-containing fibrils. More recently, Carraher and Schwarzbauer [50] utilized a polyacrylamide model to evaluate the role of matrix stiffness on fibronectin organization. Polyacrylamide scaffolds have become popular three-dimensional models as their rigidity can be modulated by altering the ratios of the components contributing to polymerization of the scaffold. Similar to previous studies, this work illustrated that growth of cells on more rigid substrates promoted fibronectin assembly and activation of focal adhesion kinase (FAK). Furthermore, activation of ECM receptors of the integrin family by Mn^2+^ on softer substrates stimulated fibronectin assembly illustrating that integrin activity is an important mediator of this process (discussed further below). Previous studies have illustrated that the conformation of fibronectin on more rigid substrata is extended, which exposes additional binding sites for cells to fibronectin [51]. This is consistent with other studies illustrating that multiple proteins that are involved in mechanotransduction become extended in response to mechanical force thus revealing cryptic interaction sites that mediate activity of the proteins. Indeed, providing exogenous unfolded fibronectin to cells in “soft” polyacrylamide gels increases FAK activation to a similar degree as culture in more rigid gels [50]. 

## 4. ECM Density and Myofibroblast Formation

An important step in tissue fibrosis of many organs is the formation of myofibroblasts or myofibroblast-like cells. These cells are characterized by enhanced contractile activity, formation of stress fibers, and expression of *α*-smooth muscle actin. Myofibroblasts are responsible for alterations to connective tissues including increased synthesis of ECM components. In addition, these cells produce cytokines and growth factors that promote the fibrotic response in an autocrine/paracrine manner. Myofibroblasts are derived from a variety of cells in response to tissue damage and stress including quiescent fibroblasts, blood-derived fibrocytes, mesenchymal stem cells, stellate cells of the liver, and others [52, 53]. Regardless of their origin, myofibroblasts likely arise as an acute and beneficial response to repair damaged tissue. Continued myofibroblast contraction and production of ECM components become deleterious and in many cases yield to stiff fibrotic tissue that obstructs and destroys organ function [54]. Stiffened tissue further promotes myofibroblast formation perpetuating scar formation. 

Studies using a three-dimensional collagen scaffold system illustrated that collagen deformability or compliance is inversely related to the transformation of cells into a myofibroblast phenotype [55]. Culture of cells on plastic coated with thin films of collagen (minimal compliance and maximal generation of intracellular tension) resulted in the highest levels of *α*-smooth muscle actin expression, routinely used as a marker for myofibroblast formation. Culture of cells in free-floating collagen gels (maximal compliance and least generation of intracellular tension) yielded the lowest relative level of *α*-smooth muscle actin expression. Similar results have been obtained in experiments examining matrix rigidity and differentiation of bronchial fibroblasts to a myofibroblast phenotype [56]. Culture of bronchial fibroblasts on polydimethylsiloxane substrates of variable stiffnesses (1–50 kPa) was performed to evaluate the effects of matrix mechanical properties on myofibroblast formation [56]. Increased scaffold stiffness promoted myofibroblast formation and increased *α*-smooth muscle actin and interstitial collagen expression. In the former studies, the expression of the *α*1 and *α*2 integrins, which are collagen receptors, correlated to enhanced myofibroblast formation on collagen-coated plastic [55]. Incubation of cells with function-blocking antibodies to these integrins attenuated myofibroblast formation indicating that generation of intracellular tension via integrin-ECM interactions is critical to the transformation process. More recent studies have illustrated an interaction between the mechanical properties of three-dimensional collagen gels and the biochemical environment [57]. In these studies, there was no difference in *α*-smooth muscle actin expression between cells in free-floating and constrained collagen gels cultured in low serum (5%); however, enhanced *α*-smooth muscle actin expression was seen in constrained gels at higher serum levels (10%). These studies and others illustrate integration of mechanical and biochemical signals by cells.

The conversion of hepatic stellate cells to a myofibroblast phenotype is a critical step in liver fibrosis and is part of the pathway to cirrhosis in chronic liver disease. Culture of hepatic stellate cells on tissue culture plastic and in high levels of serum results in their spontaneous conversion to a myofibroblast phenotype [58]. Culture of hepatic cells on Matrigel, a relatively soft basement membrane-like matrix, retains the quiescent nature of hepatic stellate cells [59]. Furthermore, culture of differentiated hepatic myofibroblasts on Matrigel results in loss of myofibroblast characteristics [60]. The mechanisms of the dedifferentiation of these cells are not well understood. Recent studies by Olsen et al. [61] to evaluate the role of substrate stiffness on differentiation of hepatic stellate cells utilized polyacrylamide scaffolds coated with various ECM substrates. These studies illustrated that increased matrix stiffness is capable of promoting myofibroblast formation independent of growth factor or cytokine stimulation. However, addition of TGF-*β* to the culture medium enhanced differentiation on stiff scaffolds, again indicating interactions between the mechanical and biochemical environments. These studies also illustrated that interactions between the cells and the surrounding ECM and generation of mechanical tension are critical to the conversion to a myofibroblast phenotype. That is, coating of polyacrylamide scaffolds with collagen or fibronectin promoted myofibroblast formation to a much greater degree than polyacrylamide scaffolds coated with poly-L-lysine. Cell adhesion to poly-L-lysine is through electrostatic charges and not via specific integrin receptors. Studies with foreskin fibroblasts have illustrated that alterations in integrin expression accompany changes in substrate rigidity and myofibroblast formation [62]. In these studies, cells cultured on less rigid polyacrylamide gels expressed little *α*-smooth muscle actin and primarily the *α*2*β*1 integrin. Culture of cells on more rigid substrata resulted in enhanced expression of *α*-smooth muscle actin and a switch to expression primarily of *α*v*β*3 integrin.

Fibroblasts isolated from diseased patients or animal models typically retain characteristics of their altered phenotype *in vitro* [63]. Indeed, comparison of fibroblasts from normal individuals and individuals with idiopathic pulmonary fibrosis illustrated differences in proliferation and contractile activity on rigid substrates [64]. However, the fibroblasts from idiopathic pulmonary fibrosis patients remained responsive to alterations in matrix rigidity with decreased proliferation and contractile properties when plated in soft matrices. This suggests that the myofibroblast phenotype is not a permanent state but can be reversed by alterations in the matrix properties. In contrast to this, studies culturing fibroblasts for prolonged periods on matrices of different mechanical properties suggest the conversion to a myofibroblast phenotype is a more “permanent” condition [65]. Culture of cells on a rigid matrix for three weeks resulted in sustained fibrotic activity, even after moving the cells to softer matrices. Understanding the plasticity of the fibrotic phenotype is critical to development of novel therapeutic approaches to fibrosis.

Recent studies have been carried out utilizing a novel photodegradable cross-linker-polyethylene glycol scaffold in which exposure to ultraviolet light can modulate the mechanical properties of the substratum to evaluate the effects on myofibroblast conversion of heart valve interstitial cells [66]. Similar to studies with other cell types, increased elastic modulus of the scaffold yielded an enhanced proportion of *α*-smooth muscle actin-containing cells. Interestingly, and of potential therapeutic significance, the proportion of myofibroblasts in the scaffolds decreased by approximately half when the elastic modulus was decreased by photodegradation. This coincided with a reduction in connective tissue growth factor and in proliferation. The classic dogma has been that once fibrosis has begun, it cannot be reversed; however, recent studies have illustrated that fibrosis can be halted or even reversed depending upon the extent of its progression [67]. The above studies suggest that alteration in the ECM biomechanical properties may be an important therapeutic target that is able to modulate myofibroblast formation and fibrosis.

Recent studies with gold nanoparticles have shown that they can be used for both measuring cell-induced deformation of the ECM as well as modulating matrix stiffness and formation of myofibroblasts. Stone et al. [68] described a method using the light scattering properties of gold nanorods as a pattern marker to track cardiac fibroblast deformation of a two-dimensional collagen matrix using digital image correlation. This study detected areas of both tensile and compressive strain within the collagen films and displacements on the order of 18 *μ*m [68]. Recently this method was applied to examine age-dependent differences in cellular mechanical behavior. Cardiac fibroblasts isolated from neonatal and adult rats were examined for their ability to deform a two-dimensional collagen film and three-dimensional collagen gels [69]. While no significant differences in strain were detected between the cell populations on the two-dimensional films, neonatal fibroblasts were significantly more contractile in three-dimensional collagen gels and expressed higher levels of *α*-smooth muscle actin compared to adult fibroblasts. Inclusion of negatively charged, polyelectrolyte-coated gold nanorods within three-dimensional collagen gels significantly reduced the ability of neonatal cardiac fibroblasts to contract these gels and was accompanied by a significant decrease in both the expression of *α*-smooth muscle actin and type I collagen [70]. This study suggested that the presence of the surface-modified nanorods impaired the ability of the fibroblasts to transform into myofibroblasts. In addition, it has been shown that negatively charged nanorods accelerated the *in vitro* assembly to type I collagen, and rheological characterization of the mechanical properties of these constructs revealed that these gels were stiffer and more elastic than controls or gels containing positively charged gold nanorods [71]. These latter studies would suggest that nanomaterials may hold promise as a means to both alter the mechanical properties of the ECM and the formation of the myofibroblast phenotype associated with pathological fibrosis.

Another mechanism to take advantage of matrix mechanical properties therapeutically is in targeting death of cells via alterations in matrix rigidity. It has long been known that interactions with the ECM are necessary for survival of normal cells. However, the effects of the mechanical properties of the ECM on cell survival are only recently being addressed. Using polyacrylamide gels of varying rigidity coated with type I collagen, Wang et al. [72] illustrated that proliferation of NIH 3T3 cells is enhanced on stiffer scaffolds. These studies also illustrated that apoptosis of NIH 3T3 cells was increased by almost two fold on less rigid collagen-coated polyacrylamide gels. The effect of matrix stiffness on apoptosis was absent in H-ras-transformed cells. A similar increase in apoptosis was seen in cells from the rat annulus fibrosis when cultured on softer polyacrylamide scaffolds [73]. These studies suggest that decreasing local matrix stiffness will result in apoptosis, potentially of matrix-producing myofibroblasts or other cells.

The ability of matrix mechanical properties to direct cell behavior is also being integrated into novel tissue engineering approaches, particularly in attempting to develop vascularized tissue constructs [74]. Examination of the invasive activity of endothelial cells plated onto the surface of collagen scaffolds has been used as an angiogenic model. Increasing the stiffness of the collagen scaffolds by cross-linking with microbial transglutaminase resulted in increased numbers of angiogenic sprouts and enhanced cell invasion independent of ECM pore size or density [75]. Under the appropriate biochemical and mechanical conditions, endothelial cells are able to form three-dimensional networks. Utilizing polyacrylamide gels functionalized with peptide sequences derived from cell adhesion sequences, the effect of scaffold mechanical properties on network formation was evaluated [76]. Endothelial cells formed stable networks on relatively soft functionalized polyacrylamide gels (Young's modulus of 140 Pa) in the absence of angiogenic biochemical factors (bFGF or VEGF). On stiffer polyacrylamide scaffolds (2500 Pa), endothelial cells failed to assemble into networks in the presence or absence of angiogenic factors. Thus, the elastic modulus of hydrogels is able to direct the migration and organization of vascular cells [74].

## 5. Transduction of Mechanical Signals

Studies utilizing *in vitro* systems have provided fundamental information regarding the molecular mechanisms whereby cells detect and respond to mechanical forces. During the past two decades, extensive progress has been made in understanding “mechanotransduction” or the mechanisms whereby physical stimuli are converted into chemical signals by cells [77, 78]. Despite the fact that the types of mechanical forces cells experience are variable, including externally applied forces (stretch, shear stress, compression, etc.) and forces generated by cells themselves, the molecular mechanisms whereby this information is transduced appear to have similarities. Alterations in the three-dimensional conformation of mechanosensitive proteins or adhesion structures are often at the foundation of this process. Studies utilizing mechanical stretch systems were fundamental in implicating cell surface integrins as central components of cell adhesion complexes and fundamental to mechanotransduction [79]. Integrins are heterodimers composed of an alpha and a beta chain that serve as the primary family of receptors for ECM components [80–82]. There are over twenty different *α*/*β* heterodimer combinations, and specific *α*/*β* heterodimers serve as receptors for particular ECM ligand(s). The response of cells to mechanical stretch varies depending upon the ECM substratum suggesting a role for specific integrin heterodimers [79, 83]. Utilizing function-blocking antibodies to specific integrins (*α*4 and *α*5 chains) or arginine-glycine-aspartic acid (RGD) peptides to prevent integrin-ECM interactions, MacKenna et al. [79] were among the first to show roles for specific integrins in the response of fibroblasts to mechanical stretch.

**Figure 1:**
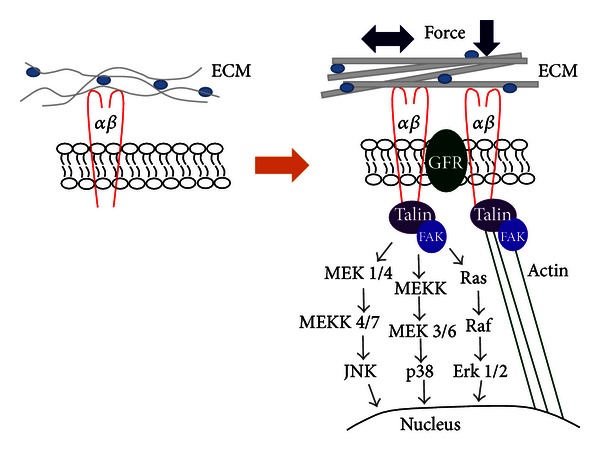
This schematic illustrates the transduction of mechanical force from the microenvironment to the cell. Extrinsically applied force results in alteration in the three-dimensional structure of the ECM and activation of integrin-associated signaling and transmission of signals via the actin cytoskeleton. These forces subsequently result in accumulation of ECM components and a stiffer ECM, which exacerbates the fibrotic response.

These early studies set the stage for extensive research focused on the mechanisms whereby cells detect mechanical changes in the microenvironment and transduce these into biochemical and molecular alterations in the cytoplasm and nucleus. The cell-ECM linkage involving integrins and a myriad of associated proteins is a critical component of this process (Figure 1). It has become increasingly clear that integrin-based adhesions are dynamic and complex structures that transmit information from the ECM to the cell and vice versa [84]. Integrins, which lack intrinsic enzyme activity, provide a physical linkage from the ECM to the actin cytoskeleton and to a wide array of signaling proteins. In fact, integrin complexes can contain over a hundred different proteins, many that bind in a force-dependent manner [85, 86]. The characterization of the ECM-integrin-cytoskeletal linkage has contributed to the concept of tensegrity in which signals can be transmitted from the ECM to the cytoplasm and nucleus via these physical connections [87, 88]. Several proteins can simultaneously bind integrins and actin and are thus thought to participate in mechanotransduction via the physical ECM-integrin-cytoskeleton linkage including vinculin, talin, and *α*-actinin [89, 90].

A number of signaling molecules associate directly or indirectly with the integrin cytoplasmic domain including focal adhesion kinase (FAK). FAK was initially identified as a Src kinase substrate [91, 92]. As integrins do not have intrinsic enzyme activity, FAK is a critical mediator of integrin-induced signaling events. The activation of FAK is initiated by autophosphorylation of tyrosine at position 397 and can be induced by clustering of integrins [93, 94]. In turn, FAK can activate integrins, which strengthens cell adhesions with the ECM [95]. Activated FAK can act independently or as part of a Src-containing complex to phosphorylate other signaling proteins or act as a scaffold in the recruitment of additional proteins to cell adhesions.

Exposure of cells to mechanical force results in activation of numerous intracellular signaling pathways including protein kinases such as protein kinase C, c-Jun N-terminal kinases (JNK), extracellular signal-regulated kinases (Erk), and others (see [96] for recent review). Activation of these pathways ultimately leads to activation of transcription factors and cell activities that comprise the response of a given cell to mechanical events.

While there appear commonalities in signaling pathways induced by various types of mechanical forces, *in vitro* studies illustrate that cells respond differently to diverse types of mechanical perturbations. The type of mechanical force can modulate differentiation of connective tissue cells. The ratio between tensile and compression type forces can promote either differentiation into cartilage or bone [97]. Exposing heart fibroblasts to constant versus cyclic mechanical stretch resulted in differences in collagen gene expression [98]. Similarly, exposing vascular endothelial cells to cyclic stretch resulted in differences in growth factor expression and branch formation compared to constant stretch [99]. Application of steady mechanical force on aortas resulted in more pronounced FAK activation compared to pulsatile stretch [100]. These studies suggest that while generalities may be developed regarding the response of cells to mechanical force, the details of this response likely vary depending on the type of force and in a cell- or tissue-specific manner. 

## 6. YAP/TAZ as Mechanotransducers

Recent studies have illustrated that signals from the ECM and cell adhesion sites converge on two components of the Hippo pathway, Yes-associated protein (YAP) and transcriptional coactivator with PDZ-binding motif (TAZ) [101, 102]. Analysis of the expression of YAP and TAZ illustrated that the levels of these proteins were enhanced in endothelial cells cultured on stiff fibronectin-containing polyacrylamide hydrogels (10–40 kPa) compared to cells growing on soft hydrogels (0.7–1.0 kPa) [101]. The expression of YAP and TAZ on stiff hydrogels was similar to that seen in cells cultured on plastic culture dishes. In addition, the subcellular localizations of YAP and TAZ are altered by the ECM mechanical environment. These proteins are predominantly located in the cytoplasm of cells grown in softer matrices but are translocated to the nucleus in cells cultured in stiff substrates. YAP and TAZ modulate the activity of transcription factors, including LEAD, RUNx, and Smads in the nucleus. Among the transcriptional targets of the YAP and TAZ system are connective tissue growth factor and TGF-*β*, two important biochemical factors that promote fibrosis, and transglutaminase-2, an important component of ECM deposition and turnover [103]. 

Several recent studies have begun to evaluate the functional roles of YAP and TAZ in mediating the response of cells to mechanical forces. In humans, the trabecular meshwork of the eye is approximately twentyfold stiffer in individuals with glaucoma than in normal individuals [104]. Cells from the trabecular meshwork have been cultured on hydrogels of varying stiffness representing normal and glaucomatous conditions (5 kPa and 75 kPa, resp.) to evaluate the role of the YAP/TAZ system in the progression of fibrosis associated with glaucoma. Similar to the above studies, culture of trabecular meshwork cells on stiffer ECM resulted in enhanced expression of TAZ and transglutaminase-2. Interestingly, YAP expression was decreased relative to that on softer scaffolds suggesting that there may be cell-specific regulation of YAP and TAZ in response to altered mechanical properties of the microenvironment. 

## 7. Conclusions and Future Directions

It has become increasingly clear that most cells in the vertebrate body are exposed to varying degrees of mechanical forces. These forces impact embryonic development, homeostasis, and pathological conditions including fibrosis. Historically most of the studies that focused on mechanical force as a profibrotic stimulus utilized two-dimensional stretch or compression models with isolated matrix-producing cells. These studies have provided substantial knowledge regarding the responses of cells to mechanical force and the underlying mechanisms of this response. However, these systems do not adequately mimic the *in vivo* three-dimensional environment. This has led to development of three-dimensional models to evaluate the effects of mechanical forces in a more *in vivo*-like environment. The realization that the biomechanical properties of the microenvironment can promote fibrosis and other responses has led to renewed interest in the effects of mechanical forces on cell and tissue behavior.

While extensive knowledge has been gained regarding the effects of the mechanical environment on cells and tissues, many questions remain regarding the molecular mechanisms of these effects. Identification of novel mechanoresponsive proteins such as YAP and TAZ will provide new therapeutic targets to modulate the deleterious effects of increased mechanical force. As it is becomingly increasing clear that tissue stiffness may precede fibrosis or at least contribute to ongoing fibrosis, identifying methods to modulate the mechanical properties of the microenvironment may also yield novel therapeutic approaches. Along these lines, specific nanomaterials may provide such reagents. However, the mechanisms whereby these materials regulate tissue properties have not been elucidated.
